# Microglia positron emission tomography and progression in multiple sclerosis: thalamus on fire

**DOI:** 10.1093/braincomms/fcaf141

**Published:** 2025-04-16

**Authors:** Burcu Zeydan, Nur Neyal, Jiye Son, Christopher G Schwarz, June C Kendall Thomas, Holly A Morrison, Melissa L Bush, Robert I Reid, Scott A Przybelski, Angela J Fought, Clifford R Jack, Ronald C Petersen, Kejal Kantarci, Val J Lowe, Laura Airas, Orhun H Kantarci

**Affiliations:** Department of Radiology, Mayo Clinic, Rochester, MN 55905, USA; Women’s Health Research Center, Mayo Clinic, Rochester, MN 55905, USA; Department of Radiology, Mayo Clinic, Rochester, MN 55905, USA; Department of Radiology, Mayo Clinic, Rochester, MN 55905, USA; Department of Radiology, Mayo Clinic, Rochester, MN 55905, USA; Department of Radiology, Mayo Clinic, Rochester, MN 55905, USA; Center for Multiple Sclerosis and Autoimmune Neurology, Mayo Clinic, Rochester, MN 55905, USA; Center for Multiple Sclerosis and Autoimmune Neurology, Mayo Clinic, Rochester, MN 55905, USA; Department of Radiology, Mayo Clinic, Rochester, MN 55905, USA; Department of Quantitative Health Sciences, Mayo Clinic, Rochester, MN 55905, USA; Department of Quantitative Health Sciences, Mayo Clinic, Rochester, MN 55905, USA; Department of Radiology, Mayo Clinic, Rochester, MN 55905, USA; Department of Neurology, Mayo Clinic, Rochester, MN 55905, USA; Department of Radiology, Mayo Clinic, Rochester, MN 55905, USA; Women’s Health Research Center, Mayo Clinic, Rochester, MN 55905, USA; Department of Radiology, Mayo Clinic, Rochester, MN 55905, USA; Turku PET Center and Division of Clinical Neurosciences, University of Turku, Turku 20521, Finland; Neurocenter, Turku University Hospital, Turku 20521, Finland; Center for Multiple Sclerosis and Autoimmune Neurology, Mayo Clinic, Rochester, MN 55905, USA; Department of Neurology, Mayo Clinic, Rochester, MN 55905, USA

**Keywords:** multiple sclerosis, thalamus, microglia, ^11^C-ER176 positron emission tomography, magnetic resonance imaging

## Abstract

Increased innate immune activity promotes neurodegeneration and contributes to progression in multiple sclerosis. This prospective case-control study aims to investigate thalamic microglia density on 18kDa translocator protein PET in patients with multiple sclerosis using a third-generation radioligand, ^11^C-ER176, and investigate the associations of ^11^C-ER176 PET uptake with imaging and clinical measures of progression in multiple sclerosis. Patients with multiple sclerosis (*n* = 50) and controls (*n* = 55) were prospectively enrolled and they underwent ^11^C-ER176 PET and MRI including diffusion MRI with neurite orientation dispersion and density imaging. Disease characteristics, expanded disability status scale and multiple sclerosis functional composite scores were obtained in patients with multiple sclerosis. Age at imaging (mean ± standard deviation: patients = 49.6 ± 12.9 years, controls = 48.2 ± 15.4 years, *P* = 0.63) and sex (female ratio; patients = 72%, controls = 65%, *P* = 0.47) were not different between the groups. Thalamus ^11^C-ER176 PET uptake was highest in patients with progressive multiple sclerosis (1.272 ± 0.072 standardized uptake value ratio), followed by patients with relapsing multiple sclerosis (1.209 ± 0.074 standardized uptake value ratio) and lowest in controls (1.162 ± 0.067 standardized uptake value ratio, *P* < 0.001). Patients with thalamic lesions had higher thalamus ^11^C-ER176 PET uptake than those without thalamic lesions in both relapsing multiple sclerosis and progressive multiple sclerosis (*P* < 0.001). In patients with multiple sclerosis, higher thalamus ^11^C-ER176 PET uptake correlated with lower thalamic volume (*r* = −0.45, *P* = 0.001), higher mean diffusivity (*r* = 0.56, *P* < 0.001), lower neurite density index (*r* = −0.43, *P* = 0.002), lower orientation dispersion index (*r* = −0.40, *P* = 0.005) and higher free water fraction (*r* = 0.42, *P* = 0.003) in the thalamus. In patients with multiple sclerosis, higher thalamus ^11^C-ER176 PET uptake also correlated with higher mean diffusivity (*r* = 0.47, *P* < 0.001) and lower neurite density index (*r* = −0.36, *P* = 0.012) in the corpus callosum. In patients with multiple sclerosis, higher thalamus ^11^C-ER176 PET uptake correlated with worse expanded disability status scale scores (*r* = 0.33, *P* = 0.02), paced auditory serial addition test scores (*r* = −0.43, *P* = 0.003) and multiple sclerosis functional composite *z*-scores (*r* = −0.46, *P* = 0.001). Microglia density in the thalamus is highest in patients with progressive multiple sclerosis and is associated with imaging biomarkers of neurodegeneration and clinical disease severity. As a signature imaging biomarker of progression in multiple sclerosis, effectively reflecting the global disease burden, ^11^C-ER176 PET may aid development and efficacy evaluation of therapeutics targeting microglia.

## Teaser

Zeydan *et al*. report that thalamic microglia density on ^11^C-ER176 PET is highest in progressive multiple sclerosis, followed by relapsing multiple sclerosis and controls. As a molecular imaging biomarker of progression in multiple sclerosis, ^11^C-ER176 PET uptake in the thalamus correlates with MRI biomarkers of neurodegeneration and clinical disease severity in multiple sclerosis.

## Graphical Abstract

**Graphical Abstract:**
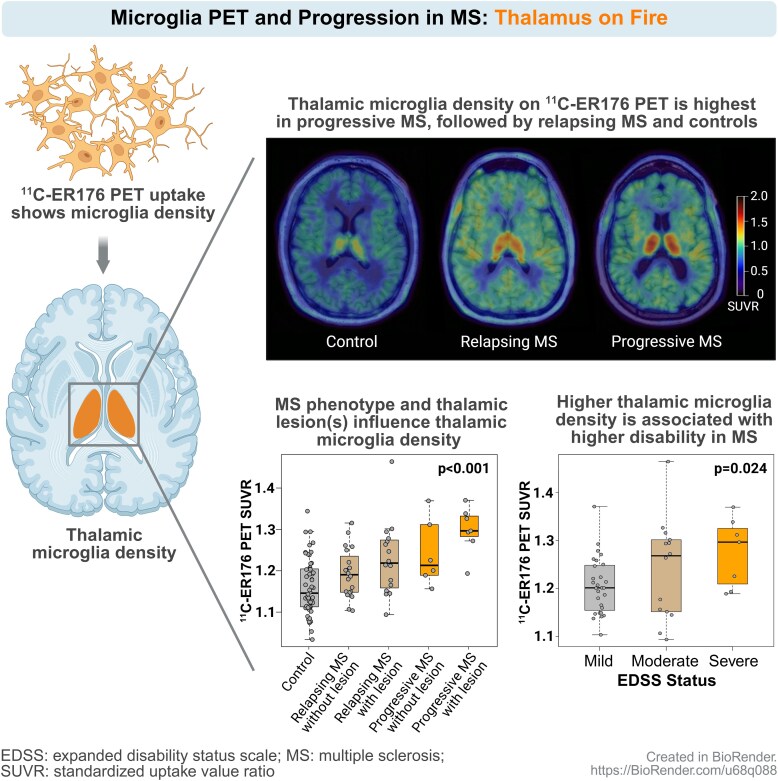

## Introduction

Increased innate immune activity and associated reactive microglia within the central nervous system (CNS) promote neurodegeneration and contribute to the development of progression in multiple sclerosis (MS).^1^  ^11^C-ER176 is a third-generation 18kDa translocator protein (TSPO) PET radioligand that targets glia and can reliably quantify persistent microglia density in the brain.^2^

Brain MRI is commonly used to detect volumetric changes in MS. Cortical thickness is a typical area of interest for evaluating structural changes in the gray matter (GM). The corpus callosum, a key interhemispheric relay region, serves as a dense purely white matter (WM) bundle for evaluating structural changes in the WM on MRI in MS. In addition to these important regions of neurodegeneration, the thalamus, a critical deep GM relay of the CNS, is another commonly affected region in MS. Volumetric changes in the thalamus assessed by MRI provide valuable insights into the underlying early tissue loss that is reflective of disease burden on the extensive network of connections in the CNS. Thalamic volume change occurs very early in the disease course in patients with MS (pwMS).^3^ While having lower resolution than MRI, PET imaging can detect subtle changes due to specific disease mechanisms at a molecular level, such as increased innate immune activity,^4^ which is closely related to early progression in MS.^1^  ^11^C-ER176 PET in the thalamus, therefore, has the potential to identify subtle innate immunity-related pathological changes *in vivo* earlier than MRI in pwMS.

Progressive MS treatment continues to be challenging, and the development of new treatment options is ongoing, including therapeutics targeting microglia. Bruton’s tyrosine kinase (BTK) inhibitors affect both adaptive and innate immune systems,^5^ and microglia are the main source of cellular BTK expression in the CNS.^6^ Therefore, ^11^C-ER176 PET emerges as a promising candidate outcome measure in clinical trials on progressive MS and therapeutics targeting microglia including BTK inhibitors, especially if a singular regional (e.g. thalamus) microglia density can be identified as a practical imaging metric, which associates with clinical phenotype, disease spectrum and disability in pwMS.

In this prospective case-control study, we hypothesized that thalamus would show the highest ^11^C-ER176 PET uptake compared with all other deep and cortical GM regions of the brain. Therefore, we aimed to (i) compare ^11^C-ER176 PET uptake among controls, relapsing pwMS and progressive pwMS, (ii) determine the influence of age and sex on ^11^C-ER176 PET uptake, and (iii) investigate the associations of ^11^C-ER176 PET uptake with biomarkers of neurodegeneration using MRI, including diffusion MRI with neurite orientation dispersion and density imaging (NODDI) and with clinical metrics of disability in pwMS.

## Methods

### Study population

Individuals with MS (*n* = 50), who were evaluated in the Mayo Clinic, Rochester MS clinic, were prospectively enrolled in the Mayo Clinic Advanced Imaging in Multiple Sclerosis (MCAIMS) study between August 2023 and July 2024. Control participants (*n* = 55) who did not have MS were prospectively recruited from the community that responded to the study advertisements and from the Mayo Clinic Study of Aging, which is a prospective population-based study.^7^ The controls were balanced on age and sex to the MS group.

The MS diagnosis was confirmed using the MS diagnostic criteria.^8-10^ Individuals who had any type of CNS involvement/disease were not eligible for the control group. Individuals younger than 18 years of age and/or who used methylprednisolone or prednisolone within 2 weeks before MRI/PET acquisition were not eligible to be enrolled in the study as a control or a pwMS.

Each participant underwent a 3.0 Tesla brain MRI and a ^11^C-ER176 PET/CT. PwMS completed expanded disability status scale (EDSS)^11^ (evaluated in three groups: group 1-mild = EDSS ≤2.5, group 2-moderate = EDSS 3.0–5.5, group 3-severe = EDSS ≥6.0), and Multiple Sclerosis Functional Composite (MSFC)^12^ including its three components: 25-foot walk test (25FWT) assessing leg function, 9-hole peg test (9HPT) assessing hand/arm function and paced auditory serial addition test (PASAT) assessing cognitive function. Data on disease onset, progressive MS onset, disease phenotype and disease-modifying therapy (DMT) were extracted in the MS group.

The study protocol was approved by the Mayo Clinic Institutional Review Boards, and each participant signed informed consent.

### MRI methods

MRI was performed on a 3.0 Tesla scanner (Prisma, Siemens Healthcare, Erlangen, Germany, XA30 software and 64-channel head coil). MRI protocol included a T1-weighted 3D high resolution magnetization–prepared rapid gradient-echo (MPRAGE) sequence for anatomical segmentation and labelling. MRIs were segmented, and GM volume and cortical thickness were measured using a previously published pipeline based on SPM12, ANTs, and the Mayo Clinic Adult Lifespan Template (MCALT, https://www.nitrc.org/projects/mcalt/).^13,14^ Volumetric analysis was adjusted for total intracranial volume and presented as percentage. Cortical thickness meta-ROI was calculated by averaging the mean thickness across all cortical regions.

All diffusion MRI images were acquired with a spin echo single shot Echo Planar Imaging sequence with 2.0 mm isotropic voxels. The diffusion gradients in each shell were evenly spread using an electrostatic repulsion scheme,^15^ modified to distribute them over whole spheres instead of hemispheres. After denoising the images,^16^ head motion and eddy current distortion were corrected using FSL’s eddy programme.^17^ Diffusion tensors were estimated using nonlinear least squares fitting and were used to calculate fractional anisotropy (FA) and mean diffusivity (MD) images in dipy.^18^ Thalamus FA was not measured because thalamus is primarily a subcortical GM structure and not a WM tract with a directional orientation. Images of the neurite density and orientation dispersion indices [neurite density index (NDI), orientation dispersion index (ODI) and free water fraction (FWF)^19^] were calculated using the AMICO implementation.^20^ ODI in the corpus callosum was not measured because the AMICO implementation of NODDI is insensitive to small changes in ODI when the neurites are highly aligned.

### PET methods

The PET imaging was performed on a PET/CT scanner (Siemens or GE Healthcare), which operated in 3D mode using ^11^C-ER176, a third-generation radioligand (IND#:149229). PET scans were acquired in 60–80 min post-injection using four 5-min dynamic frames after an injection of ^11^C-ER176 (518 MBq, 14 mCi; range, 370–666 MBq, 10–18 mCi) and an uptake period of 60 min. The mean injected mass radioactivity was 9.5 µg. The typical specific activity at injection was 0.6 Ci/mol. PET data were reconstructed using a 3D iterative reconstruction algorithm (ordered subset expectation maximization-OSEM). Standard corrections for random coincidences, radioactive decay and attenuation were applied.

For image quantification, an automated image processing pipeline was utilized.^21^ Each participant’s own T1-weighted MRI was registered to PET images for GM, WM and atlas segmentation. ^11^C-ER176 PET uptake was not different between pwMS and controls in the cerebellum crus [pwMS = 1.006 ± 0.011 standardized uptake value ratio (SUVR), controls = 1.003 ± 0.010 SUVR, *P* = 0.141]. Furthermore, within pwMS, the ^11^C-ER176 PET uptake did not differ among pwMS with crus lesion(s) versus without crus lesions in the left or right side of the crus (*P* = 0.35 and *P* = 0.34, respectively). Therefore, the reference region of cerebellar crus uptake was used to calculate SUVRs. Previous studies have shown that having low-affinity binding genotype for TSPO is uncommon (10%),^22^ and ^11^C-ER176 binding is sensitive in all genotypes (high-, mixed- or low-affinity binding).^2,23-25^ Regardless, in the 27 random participants whose DNA samples were available in our study, the rs6971 single nucleotide polymorphism analysis showed only two participant with low-affinity binding genotype for TSPO.

### Statistical analysis

Means and standard deviations (SDs) for continuous variables and counts and percentages for categorical variables were used to summarize demographic, clinical and imaging characteristics of controls and pwMS. To compare the control, relapsing MS and progressive MS groups, *t*-tests were used for continuous variables, and chi-squared tests were used for categorical variables. The comparison of controls with relapsing and progressive MS groups used an ANOVA with Tukey Honest Significant Difference for *post hoc* pairwise comparisons. Regional ^11^C-ER176 SUVRs were analysed with AUROC for distinguishing pwMS from controls and a *t*-test for group mean differences. ANOVA and Pearson correlations were performed comparing thalamus ^11^C-ER176 SUVR with imaging and clinical variables. A trend test was performed among pwMS comparing EDSS status with thalamus ^11^C-ER176 SUVR. Lastly, due to skewness, the variables MD and FWF were analysed with a log transformation.

## Results

### Demographics and disease characteristics

Fifty pwMS and 55 control participants were prospectively enrolled. Age at imaging (mean ± SD MS = 49.6 ± 12.9 years, controls = 48.2 ± 15.4 years, *P* = 0.63) and sex (female MS = 72%, controls = 65%, *P* = 0.47) were not different between the groups.

In pwMS, age at disease onset was 35.1 ± 10.5 years. Of the 50 pwMS, 14 (28%) had progressive MS and age at progressive MS onset was 45.3 ± 8.4 years (Table 1). Age at MRI (*P* = 0.08) and sex (*P* = 0.45) were not different between relapsing and progressive pwMS.

**Table 1:** Disease characteristics in patients with MS

	Total MS *n* = 50	Relapsing MS *n* = 36	Progressive MS *n* = 14	*P-*value* Relapsing MS versus *progressive MS*
Females, N (%)	36 (72%)	27 (75%)	9 (64%)	0.449
MRI age, yrs	49.6 (12.9)	47.6 (12.9)	54.7 (11.8)	0.082
MS onset age, yrs	35.1 (10.5)	33.4 (9.5)	38.6 (11.8)	0.130
Progressive MS onset age, yrs	45.3 (8.4)	NA	45.3 (8.4)	
DMT, ever-treated, N (%)	43 (86%)	33 (92%)	10 (71%)	0.064
DMT duration, yrs	5.8 (7.4)	5.1 (6.8)	7.4 (8.9)	0.339
EDSS score, median (IQR)	1.5 (0.0–4.0)	1.0 (0.0–3.0)	5.0 (3.0–6.0)	**<0.001**
EDSS severity group, N (%)				**<0.001**
Mild (EDSS <2.5)	29 (58%)	26 (72%)	3 (21%)	
Moderate (EDSS 3.0–5.5)	14 (28%)	10 (28%)	4 (29%)	
Severe (EDSS ≥6.0)	7 (14%)	0 (0%)	7 (50%)	
25FWT score, mean (SD)	6.6 (5.9)	4.8 (1.2)	11.2 (10.0)	**<0.001**
9HPT score, mean (SD)	24.6 (13.5)	22.9 (7.9)	29.1 (22.3)	0.158
PASAT score, mean (SD)	43.9 (10.5)	44.4 (10.6)	42.6 (10.3)	0.604
MSFC *z*-score, mean (SD)	0.12 (0.58)	0.24 (0.56)	−0.19 (0.52)	**0.024**

Characteristics table of groups with the mean (SD) listed for the continuous variables [except for EDSS score where median (IQR) was used] and count (%) for the categorical variables. EDSS status: mild = EDSS <2.5, moderate = EDSS 3.0–5.5, severe = EDSS ≥6. *P-values for differences between groups come from *t*-test for continuous variables and a chi-squared test for categorical variables. Bold values indicate significance at the *P* < 0.05 level.

9HPT, 9-hole peg test; 25FWT, 25-foot walk test; DMT, disease-modifying therapy; EDSS, expanded disability status scale; IQR, interquartile range; MSFC, multiple sclerosis functional composite; PASAT, paced auditory serial addition test; SD, standard deviation.

Seven pwMS (14%) were DMT-naïve and 30 pwMS (60%) were on DMT at the time of imaging (mean DMT duration = 5.8 ± 7.4 years). Expectedly, EDSS score was higher in progressive pwMS (median, IQR 5.0, 3.0–6.0) than in relapsing pwMS (1.0, 0.0–3.0, *P* < 0.001), and severe EDSS status was more common in progressive pwMS (mild 21%, moderate 29%, severe 50%) compared with relapsing pwMS (mild 72%, moderate 28%, severe 0%, *P* < 0.001). Mean (±SD) MSFC and 25FWT scores were also expectedly worse in progressive pwMS (MSFC = −0.19 ± 0.52, 25FWT = 11.2 ± 10.0) than in relapsing pwMS (MSFC = 0.24 ± 0.56, *P* = 0.02; 25FWT = 4.8 ± 1.2, *P* < 0.001). Although 9HPT and PASAT scores were also worse in progressive pwMS than relapsing pwMS, this difference did not reach statistical significance (Table 1).

### ^11^C-ER176 PET findings

#### Patients with multiple sclerosis have higher thalamus ^11^C-ER176 PET uptake than controls

**Figure 1:**
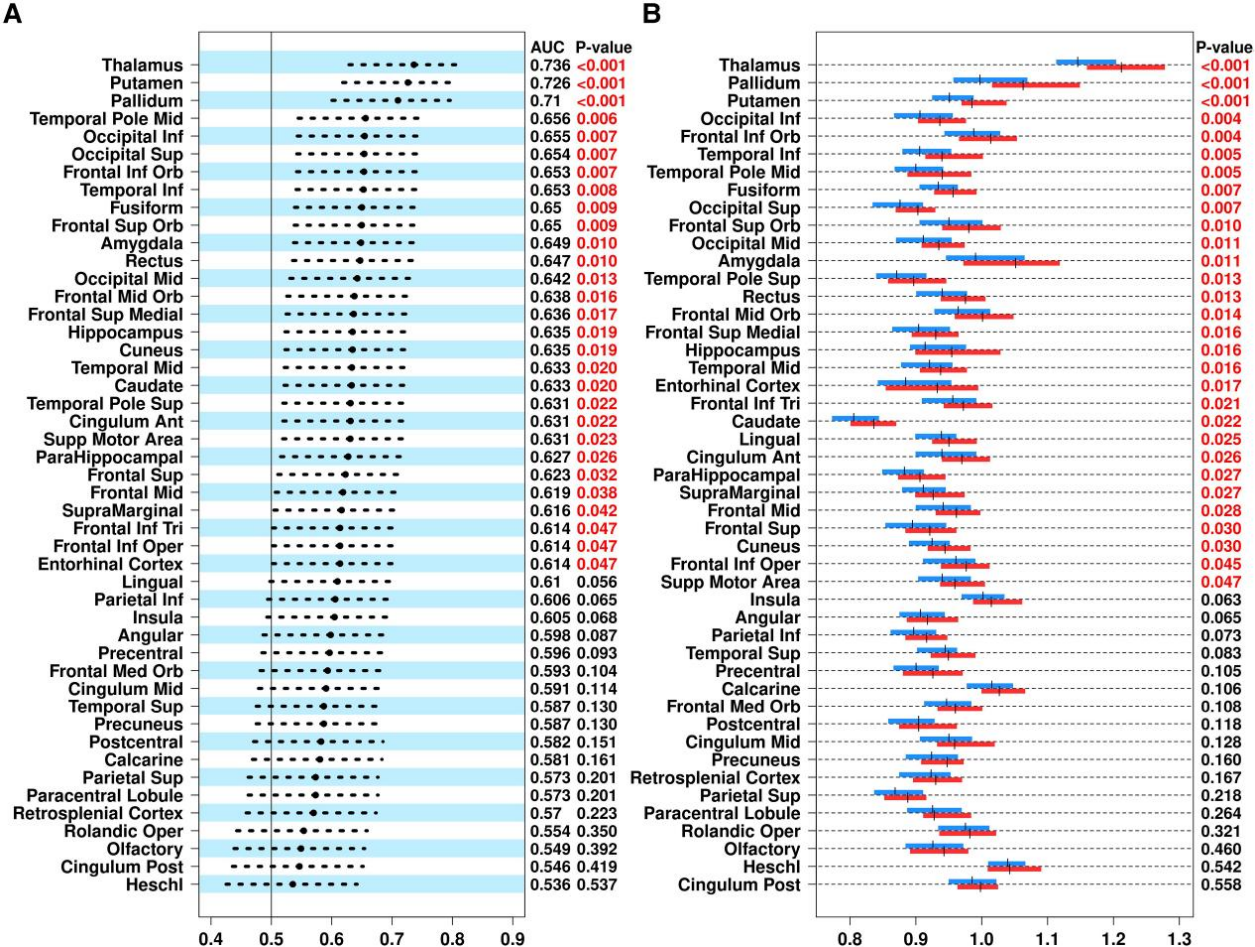
**Comparison of ^11^C-ER176 PET uptake in gray matter regions in patients with MS versus controls.** (**A**) ^11^C-ER176 PET uptake differentiated patients with MS and controls in 29 of 47 gray matter regions, and the greatest areas under the curve were seen in three deep gray matter regions: thalamus, putamen and pallidum. (**B**) ^11^C-ER176 PET uptake was higher in multiple gray matter regions in patients with MS than in controls, and it was highest in the thalamus, confirming our hypothesis that thalamus is a significant region of interest in studying MS, based on two-sample *t*-test. Both statistical tests were analysed in 54 controls and 49 patients with MS. Blue: controls, Red: patients with MS. Ant, anterior; Inf, inferior; Med, medial; Mid, middle; Orb, orbital; Oper, opercular; Post, posterior; Sup, superior; Supp, supplementary; Tri, triangular. Cingulum Ant, cingulum anterior; Cingulum Post, cingulum posterior; Frontal Inf Orb, frontal inferior orbital; Frontal Inferior Tri, frontal inferior triangular; Frontal Med Orb, frontal medial orbital; Frontal Mid, frontal middle; Frontal Mid Orb, frontal middle orbital; Frontal Sup, frontal superior; Frontal Sup Medial, frontal superior medial; Frontal Sup Orb, frontal superior orbital; Frontal Inf Oper, frontal inferior opercular; Occipital Inf, occipital inferior; Occipital Mid, occipital middle; Occipital Sup, occipital superior; Parietal Inf, parietal inferior; Parietal Sup, parietal superior; Rolandic Oper, rolandic operculum; Temporal Inf, temporal inferior; Temporal Mid, temporal middle; Temporal Pole Mid, temporal pole middle; Temporal Pole Sup, temporal pole superior; Supp Motor Area, supplementary motor area.

^11^C-ER176 PET uptake showed a significant predictive value in multiple GM regions in differentiating pwMS from controls using the AUROC analysis. Among these regions, deep GM structures including thalamus, putamen and pallidum showed the highest differentiating value between pwMS and controls (*P* < 0.001; Fig. 1A). In parallel, pwMS had higher ^11^C-ER176 PET uptake in multiple GM regions than controls with the highest ^11^C-ER176 PET uptake seen in the thalamus in both groups (controls = 1.162 ± 0.067 SUVR, MS= 1.23 ± 0.08 SUVR; *P* ≤ 0.001; Fig. 1B).

#### Progressive patients with multiple sclerosis have higher thalamus ^11^C-ER176 PET uptake than relapsing patients with multiple sclerosis

**Figure 2:**
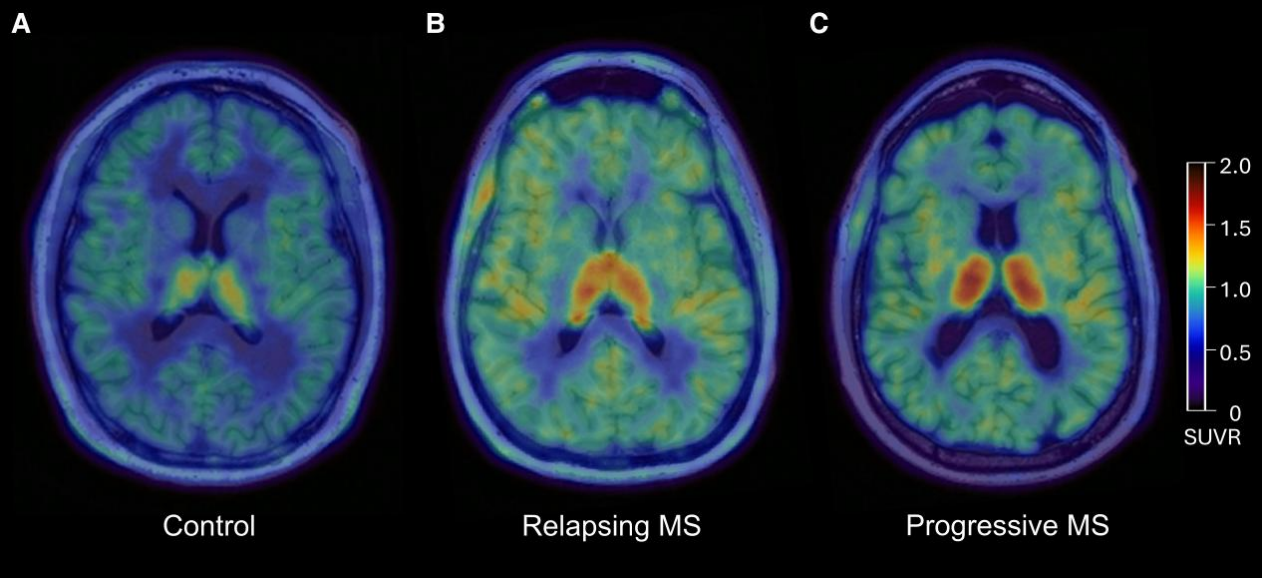
**Example ^11^C-ER176 PET images.** The images display the increase in ^11^C-ER176 PET uptake in the thalamus across a control participant, a relapsing patient with MS and a progressive patient with MS. (**A**) Thirty-year-old control male participant. Thalamus ^11^C-ER176 PET uptake was 1.034 SUVR. (**B**) Forty-two-year-old female with relapsing MS with disease onset at 32 years of age and EDSS 1.0. She was on ofatumumab at the time of PET. Thalamus ^11^C-ER176 PET uptake was 1.201 SUVR. (**C**) Fifty-six-year-old male with progressive MS with disease onset at 28 years of age, progressive MS onset at 54 years of age and EDSS 4.0. He was DMT-naïve at the time of PET. Thalamus ^11^C-ER176 PET uptake was 1.293 SUVR. Additionally, although it is not the focus of the current study, the examples illustrate that the cortical ^11^C-ER176 PET uptake is elevated in both relapsing and progressive patients with MS (**B and C**) compared with the control participant (**A**). This is also in line with the forest plots demonstrated in Fig. 1. However, the difference in the cortical ^11^C-ER176 PET uptake is not as high as the thalamus ^11^C-ER176 PET uptake, the specific focus of the current study. DMT, disease-modifying therapy; EDSS, expanded disability status scale; MS, multiple sclerosis; SUVR, standardized uptake value ratio.

**Figure 3:**
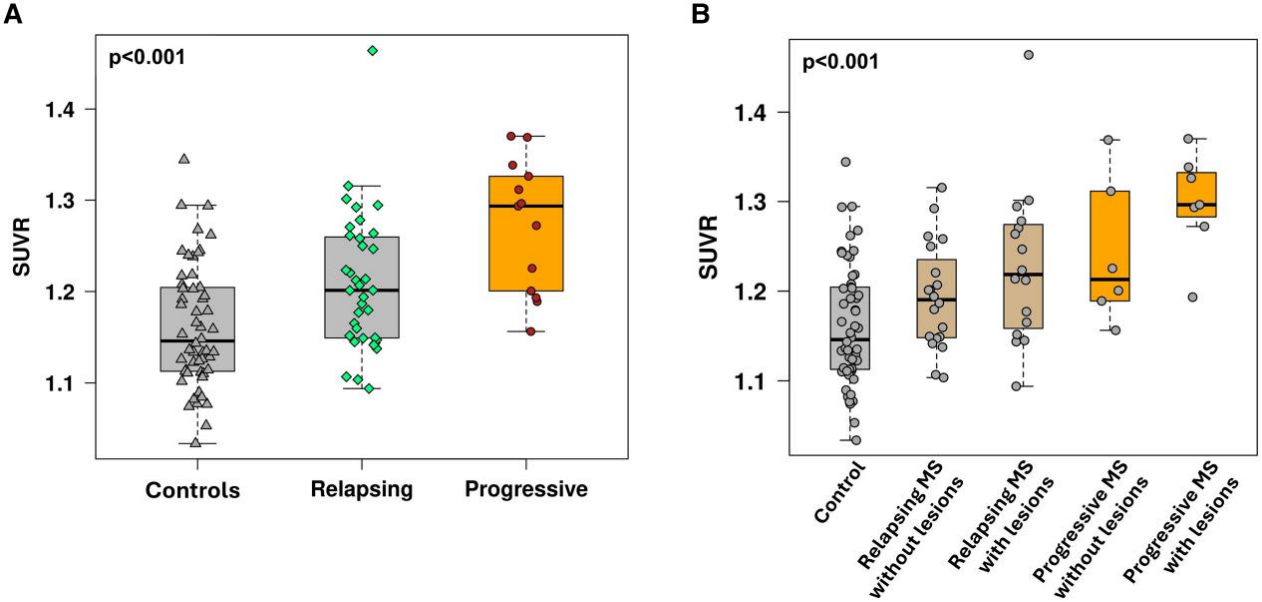
**^11^C-ER176 PET uptake across controls and MS phenotypes**. (**A**) ^11^C-ER176 PET uptake showed a gradient of increase among controls (*n* = 54), relapsing MS (*n* = 36) and progressive MS (*n* = 13) using a linear regression trend test (*P* < 0.001). It was lowest in the controls followed by relapsing MS and highest in progressive MS. (**B**) When thalamic lesion presence was taken into account, in addition to disease phenotype (relapsing or progressive), the gradient of increase in ^11^C-ER176 PET uptake persisted but with the addition of lesion impact in each group. It was lowest in the controls (*n* = 54) followed by relapsing MS without lesions (*n* = 20), relapsing MS with lesions (*n* = 16), progressive MS without lesions (*n* = 6) and highest in progressive MS with lesions (*n* = 7) using a linear regression trend test (*P* < 0.001). Data points represent ^11^C-ER176 PET SUVR in the thalamus in each participant. MS, multiple sclerosis; SUVR, standardized uptake value ratio.

Thalamus ^11^C-ER176 PET uptake was higher in progressive pwMS (1.272 ± 0.072 SUVR) than in relapsing pwMS (1.209 ± 0.074 SUVR, *P* = 0.016). Among pwMS and controls, a gradient of increase in thalamus ^11^C-ER176 PET uptake was observed (Fig. 2**)**, ranging from lowest in controls followed by relapsing pwMS to highest in progressive pwMS (*P* < 0.001; Fig. 3A).

#### PwMS with thalamic lesions have higher thalamus ^11^C-ER176 PET uptake than pwMS without thalamic lesions

PwMS were further stratified by absence (*n* = 26) or presence (*n* = 23) of thalamic lesions in addition to the disease course (relapsing versus progressive) compared with controls. Among five groups, thalamus ^11^C-ER176 PET uptake was again lowest in controls followed by relapsing MS without lesions, relapsing MS with lesions, progressive MS without lesions and highest in progressive MS with lesions (*P* < 0.001; Fig. 3B).

#### Influence of age and sex on thalamus ^11^C-ER176 PET uptake in patients with multiple sclerosis and controls

**Figure 4:**
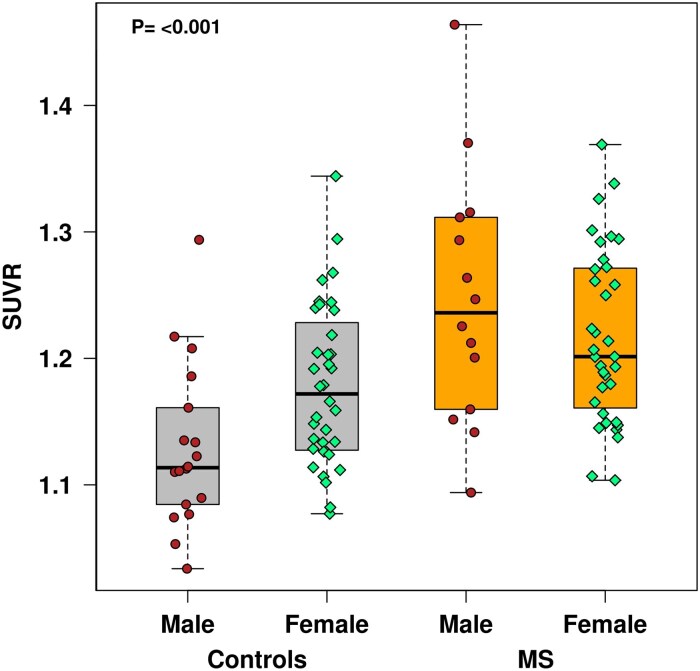
**Sex differences in ^11^C-ER176 PET uptake in controls and patients with MS.**  ^11^C-ER176 PET uptake was significantly different among male controls (*n* = 18), female controls (*n* = 36), male patients with MS (*n* = 14) and female patients with MS (*n* = 35) using an ANOVA (*P* < 0.001). Within controls, ^11^C-ER176 PET uptake was higher in females compared to males, whereas within patients with MS, it was similar between both sexes, possibly males catching up to females. Data points represent ^11^C-ER176 PET SUVR in the thalamus in each participant. MS, multiple sclerosis; SUVR, standardized uptake value ratio.

Age did not correlate with thalamus ^11^C-ER176 PET uptake in controls (*r* = 0.19, *P* = 0.17) or in pwMS (*r* = −0.05, *P* = 0.73). However, thalamus ^11^C-ER176 PET uptake showed a gradient of increase among male controls (1.13 ± 0.06 SUVR), female controls (1.18 ± 0.06 SUVR), male pwMS (1.25 ± 0.10 SUVR) and female pwMS (1.22 ± 0.07 SUVR, *P* < 0.001) (Fig. 4). Within controls, thalamus ^11^C-ER176 PET uptake was higher in females than in males (*P* = 0.009), whereas within pwMS, thalamus ^11^C-ER176 PET uptake was similar between females and males (*P* = 0.24). Within males (*P* < 0.001) and within females (*P* = 0.015), thalamus ^11^C-ER176 PET uptake was higher in pwMS than in controls.

### MRI findings

#### Patients with multiple sclerosis have greater CNS atrophy and worse white matter and neurite integrity than controls

Among controls (*n* = 55) and pwMS (*n* = 50), thalamic volume was smallest in progressive pwMS (0.371 ± 0.045), followed by relapsing pwMS (0.400 ± 0.072) and highest in controls (0.439 ± 0.056, *P* < 0.001; Table 2). Total cortical thickness was also smallest in progressive pwMS (2.852 ± 0.215 mm) followed by relapsing pwMS (2.930 ± 0.226 mm) and highest in controls (3.160 ± 0.240 mm, *P* < 0.001).

**Table 2:** Imaging characteristics in patients with MS and controls

	Controls *n* = 55	Relapsing MS *n* = 36	Progressive MS *n* = 14	*P-Value* *			
				Overall	Control versus relapsing MS	Control versus progressive MS	Relapsing MS versus progressive MS
MRI							
Cortical thickness, mm	3.160 (0.240)	2.930 (0.226)	2.852 (0.215)	**<0**.**001**	**<0**.**001**	**<0**.**001**	0.536
Thalamus ^11^C-ER176 PET, SUVR	1.162 (0.067)	1.209 (0.074)	1.272 (0.072)	**<0**.**001**	**0**.**007**	**<0**.**001**	**0**.**016**
Thalamus volume^b^	0.439 (0.056)	0.400 (0.072)	0.371 (0.045)	**<0**.**001**	**0**.**009**	**<0**.**001**	0.283
Diffusion MRI with NODDI - thalamus							
MD^a^ µm^2^/ms	0.692 (0.022)	0.696 (0.031)	0.705 (0.019)	0.162	0.682	0.144	0.432
NDI	0.475 (0.015)	0.476 (0.017)	0.468 (0.016)	0.242	0.918	0.313	0.223
ODI	0.313 (0.017)	0.311 (0.022)	0.297 (0.019)	**0**.**024**	0.874	**0**.**018**	0.060
FWF^a^	0.005 (0.009)	0.010 (0.015)	0.009 (0.006)	**<0**.**001**	**<0**.**001**	**0**.**012**	0.972
Diffusion MRI with NODDI - corpus callosum							
FA	0.706 (0.031)	0.683 (0.041)	0.670 (0.048)	**0**.**001**	**0**.**014**	**0**.**005**	0.513
MD^a^ µm^2^/ms	0.705 (0.036)	0.737 (0.053)	0.759 (0.060)	**<0**.**001**	**0**.**003**	**<0**.**001**	0.304
NDI	0.609 (0.033)	0.580 (0.052)	0.560 (0.062)	**<0**.**001**	**0**.**008**	**0**.**001**	0.366
FWF^a^	0.085 (0.015)	0.082 (0.015)	0.083 (0.011)	0.538	0.507	0.967	0.859

^*^ *P*-values are from an ANOVA with pair-wise Tukey HSD *post hoc P*-values. Bold values indicate significance at the *P* < 0.05 level.

^a^Analysed with a log transformation due to skewness.

^b^Volumetric analysis was adjusted for total intracranial volume (TIV) and presented as percentage.

FA, fractional anisotropy; FWF, free water fraction; MD, mean diffusivity; NDI, neurite density index; NODDI, neurite orientation dispersion and density imaging; ODI, orientation dispersion index; SUVR, standardized uptake value ratio.

Thalamic diffusion MRI metrics of MD and NDI were not different among controls, relapsing pwMS and progressive pwMS (*P* > 0.05). However, ODI was lowest in progressive pwMS (0.297 ± 0.019), followed by relapsing pwMS (0.311 ± 0.022) and highest in controls (0.313 ± 0.017, *P* = 0.024). Thalamic FWF was higher in progressive pwMS (0.009 ± 0.006) and relapsing pwMS (0.010 ± 0.015) compared with controls (0.005 ± 0.009, *P* < 0.001).

Corpus callosum MD was highest in progressive pwMS (0.759 ± 0.060 µm^2^/ms), followed by relapsing pwMS (MD 0.737 ± 0.053 µm^2^/ms) and controls (0.705 ± 0.036 µm^2^/ms, *P* < 0.001). Corpus callosum FA was lowest in progressive pwMS (0.670 ± 0.048), followed by relapsing pwMS (0.683 ± 0.041) and controls (0.706 ± 0.031, *P* = 0.001). Corpus callosum NDI was lowest in progressive pwMS (0.560 ± 0.062), followed by relapsing pwMS (0.580 ± 0.052) and controls (0.609 ± 0.033, *P* < 0.001). Corpus callosum FWF did not differ between the groups (*P* > 0.05).

#### Thalamus ^11^C-ER176 PET uptake correlates with MRI biomarkers of neurodegeneration in patients with multiple sclerosis

In controls, thalamus ^11^C-ER176 PET uptake did not correlate with any of the MRI biomarkers of neurodegeneration (thalamus volume; total cortical thickness; MD, NDI, ODI, and FWF metrics in the thalamus; and FA, MD, NDI, and FWF metrics in the corpus callosum; Table 3).

**Table 3:** Pearson correlations of thalamus ^11^C-ER176 PET uptake with MRI biomarkers of neurodegeneration in patients with MS and controls

^11^C-ER176 PET uptake correlation R (P*)	Controls *n* = 55	All MS *n* = 50	Relapsing MS *n* = 36	Progressive MS *n* = 14
MRI				
Cortical thickness	0.160 (0.247)	−0.070 (0.634)	0.048 (0.782)	−0.209 (0.494)
Thalamus volume	−0.040 (0.773)	**−0.450** (**0.001)**	**−0.467** (**0.004)**	−0.241 (0.428)
Diffusion MRI with NODDI - thalamus				
MD^a^	0.143 (0.312)	**0.556** (**<0.001)**	**0.609** (**<0.001)**	0.284 (0.348)
NDI	−0.208 (0.139)	**−0.431** (**0.002)**	**−0.503** (**0.002)**	−0.060 (0.845)
ODI	−0.096 (0.498)	**−0.395** (**0.005)**	**−0.360** (**0.031)**	−0.171 (0.575)
FWF^a^	0.040 (0.780)	**0.416** (**0.003)**	**0.434** (**0.008)**	0.443 (0.130)
Diffusion MRI with NODDI - corpus callosum				
FA	0.038 (0.785)	**−0.395** (**0.005)**	**−0.479** (**0.003)**	−0.158 (0.606)
MD^a^	−0.074 (0.597)	**0.471** (**<0.001)**	**0.507** (**0.002)**	0.314 (0.296)
NDI	0.123 (0.381)	**−0.357** (**0.012)**	**−0.460** (**0.005)**	−0.043 (0.888)
FWF^a^	−0.003 (0.983)	0.142 (0.329)	0.020 (0.909)	0.516 (0.071)

^a^Analysed with a log transformation due to skewness. *Bold values indicate significance at the *P* < 0.05 level.

FA, fractional anisotropy; FWF, free water fraction; MD, mean diffusivity; NDI, neurite density index; NODDI, neurite orientation dispersion and density imaging; ODI, orientation dispersion index.

However, in pwMS, higher thalamus ^11^C-ER176 PET uptake correlated with smaller thalamus volume (*r* = −0.45, *P* = 0.001) but did not correlate with total cortical thickness (*r* = −0.07, *P* = 0.63). In pwMS, higher thalamus ^11^C-ER176 PET uptake also correlated with higher MD (thalamus *r* = 0.56, *P* < 0.001; corpus callosum *r* = 0.47, *P* < 0.001), lower FA (corpus callosum *r* = −0.40, *P* = 0.005), lower NDI (thalamus *r* = −0.43, *P* = 0.002; corpus callosum *r* = −0.36, *P* = 0.012), lower ODI (thalamus *r* = −0.40, *P* = 0.005) and higher FWF (thalamus *r* = 0.42, *P* = 0.003).

#### Thalamus ^11^C-ER176 PET uptake correlates with disability and cognitive impairment in patients with multiple sclerosis

**Figure 5:**
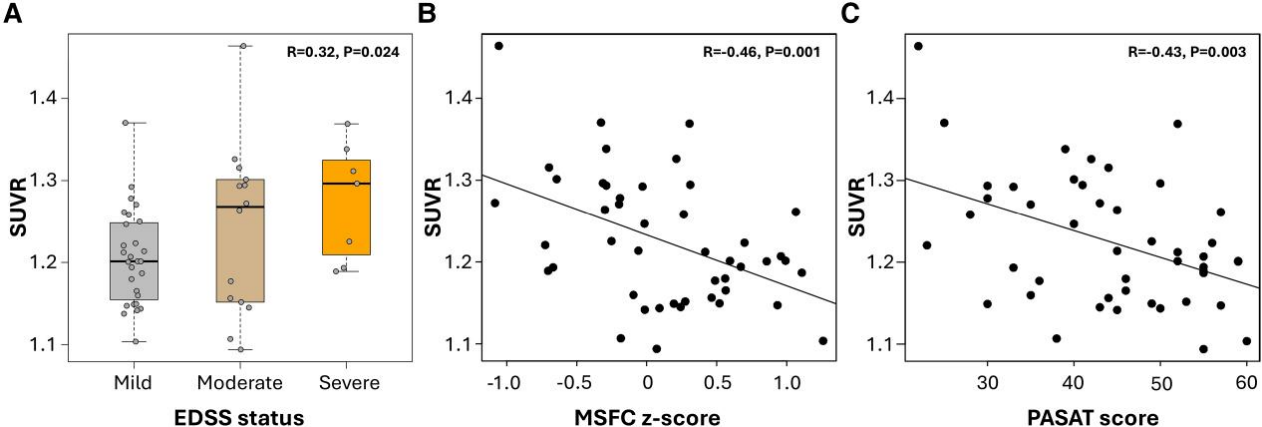
**The associations of ^11^C-ER176 PET uptake with clinical metrics in patients with MS.** Higher ^11^C-ER176 PET uptake correlated with (**A**) higher disability measured by EDSS severity [EDSS status mild-group 1 = EDSS ≤ 2.5 (*n* = 28), moderate-group 2=EDSS 3.0–5.5 (*n* = 14), severe-group 3=EDSS ≥ 6.0 (*n* = 7), *r* = 0.32, *P* = 0.024], using a linear regression trend test, (**B**) higher disability measured by MSFC *z*-score (*n* = 46, *r* = −0.46, *P* = 0.001) reporting Pearson correlations and (**C**) worse cognitive function measured by PASAT (*n* = 46, *r* = −0.43, *P* = 0.003) reporting Pearson correlations. (**A**) Data points represent thalamus ^11^C-ER176 PET SUVR for each participant. (**B**) Data points represent thalamus ^11^C-ER176 PET SUVR on the *y*-axis and MSFC *z*-score on the *x*-axis for each participant. (**C**) Data points represent thalamus ^11^C-ER176 PET SUVR on the *y*-axis and PASAT score on the *x*-axis for each participant. EDSS, expanded disability status scale; MS, multiple sclerosis; MSFC, multiple sclerosis functional composite *z*-score; PASAT, paced auditory serial addition test score; SUVR, standardized uptake value ratio.

In pwMS, higher thalamus ^11^C-ER176 PET uptake correlated with worse PASAT scores (*r* = −0.43, *P* = 0.003) and MSFC *z*-scores (*r* = −0.46, *P* = 0.001; Fig. 5), whereas it did not correlate with 25FWT scores (*r* = 0.04, *P* = 0.81) and 9HPT (*r* = 0.27, *P* = 0.07). Higher thalamus ^11^C-ER176 PET uptake also correlated with worse EDSS scores (*r* = 0.33, *P* = 0.02) and higher EDSS severity status (*r* = 0.32, *P* = 0.02). A gradient of increase in thalamus ^11^C-ER176 PET uptake was seen across the EDSS severity spectrum, lowest in mild-EDSS group 1 (EDSS ≤2.5) (1.206 ± 0.058 SUVR), intermediate in moderate-EDSS group 2 (EDSS 3.0–5.5) (1.240 ± 0.104 SUVR) and highest in severe-EDSS ≥6 group (1.275 ± 0.072 SUVR, *P* = 0.02; Fig. 5).

#### Thalamus ^11^C-ER176 PET uptake did not differ between the treatment groups in patients with multiple sclerosis

In pwMS, thalamus ^11^C-ER176 PET uptake did not differ between (i) no-DMT (1.222 ± 0.067 SUVR) versus DMT groups (1.228 ± 0.086 SUVR; *P* = 0.78) and between (ii) no-DMT versus moderate-efficacy DMT (1.221 ± 0.067 SUVR) versus high-efficacy DMT groups (1.231 ± 0.095 SUVR) (*P* = 0.91) at the time of PET. Similarly, within females (*P* = 0.78) and within males (0.53), thalamus ^11^C-ER176 PET uptake did not differ between DMT versus no-DMT groups.

## Discussion

In this prospective study of ^11^C-ER176 PET imaging, we confirmed our hypothesis that thalamus ^11^C-ER176 PET showed the greatest difference in pwMS compared with controls. Our study also provided evidence that higher density of innate immune activity in the thalamus measured by ^11^C-ER176 PET is closely associated with progressive MS and with higher disability more specifically, and it is potentially influenced by sex. We have made the following observations: (i) While many cortical and deep GM regions have elevated ^11^C-ER176 PET uptake in pwMS compared with controls, thalamus ^11^C-ER176 PET uptake was identified as the strongest GM region of interest in the brain differentiating pwMS from controls. (ii) Thalamus ^11^C-ER176 PET uptake was highest in progressive pwMS, intermediate in relapsing pwMS and lowest in controls. (iii) Both in relapsing and progressive pwMS, thalamus ^11^C-ER176 PET uptake became more pronounced when the presence of thalamic lesion(s) was considered, and the highest thalamus ^11^C-ER176 PET uptake was in progressive pwMS with thalamic lesion(s). (iv) Thalamus ^11^C-ER176 PET uptake was higher in female controls compared with male controls, but in pwMS, thalamus ^11^C-ER176 PET uptake did not differ between females and males, with possibly male pwMS catching up to female pwMS. (v) Higher thalamus ^11^C-ER176 PET uptake significantly correlated with lower thalamic volume, lower WM integrity and higher neurite damage in the thalamus and corpus callosum, and higher thalamic free water fraction as well as greater clinical disability and worse cognitive function in pwMS.

### Thalamus ^11^C-ER176 PET uptake is closely associated with having multiple sclerosis in general, and with progressive multiple sclerosis more specifically

The current study has important novel observations, and using a third-generation TSPO radioligand ^11^C-ER176 in MS is one of them. ^11^C-PK11195 is the first radioligand developed for TSPO PET and to evaluate the specificity of ^11^C-PK11195 to microglia, autoradiography and immunohistochemistry studies were conducted.^26-28^ In autoimmune encephalomyelitis and MS tissues, lesional ^11^C-PK11195 uptake correlated with activated microglial/macrophage markers,^26^ and microglia/macrophages were identified as the main cellular target of ^11^C-PK11195 binding.^27,28^ Although first- and second-generation TSPO radioligands are more widely used, the third-generation radioligand, ^11^C-ER176, which is an analogue of the first-generation ^11^C-PK11195 with a similar specificity to microglia was used in this study. The target specificity of ^11^C-ER176 has been confirmed by receptor blocking studies, the gold standard for evaluating radioligand specificity.^29^  ^11^C-ER176 has superior pharmacokinetic features over first- and second-generation radioligands, including lower lipophilicity, higher signal-to-noise ratio and higher binding affinity.^2,23-25,30^ Exclusively, ^11^C-ER176 does not generate radio-metabolites that enter the brain^29^ and has sufficient sensitivity to effectively image all three single nucleotide polymorphism rs6971 affinity genotypes *in vivo* in humans.^2,23-25^ In a study comparing four radioligands, ^11^C-ER176 showed a high specific-to-non-displaceable ratio of distribution volumes and was the only radioligand that did not require exclusion of low-affinity binders.^29^ Moreover, its signal in low-affinity binders is even higher than the signal of second-generation radioligands in high-affinity binders.^2^ Hence, ^11^C-ER176 shows the best performance in TSPO PET, and to our knowledge, ^11^C-ER176 has not been studied in MS until the current study.

In pwMS compared with controls, a higher innate immune system activity was detected on PET using ^11^C-ER176, in multiple GM regions, including the deep GM structures of thalamus, putamen, pallidum and caudate. The greatest difference in ^11^C-ER176 PET uptake between pwMS and controls was found in the thalamus. Our PET data on deep GM successfully replicated findings from a previous study demonstrating elevated TSPO PET uptake in thalamus, putamen, pallidum and caudate in pwMS, which used a completely different study population from Finland, a different radioligand (first-generation, ^11^C-PK11195) and a different quantification analysis (distribution volume ratio).^31^ Moreover, the PET signal was highest in the thalamus across all GM regions in both studies. The current study, therefore, unequivocally confirms that thalamic TSPO PET is a biomarker of overall MS pathophysiology and more specifically the progressive MS pathophysiology.

The thalamus, as a key relay of the CNS with extensive connections to other regions of the brain, is affected very early in the disease course in MS. Thalamic atrophy is present even at the presymptomatic phase of the disease.^3^ As expected, in the current study, the thalamus volume was smaller in pwMS than in controls. However, in addition to volume loss, the highest ^11^C-ER176 PET uptake was seen in the thalamus across the brain in pwMS, demonstrating the increase in CNS glial density and related innate immune activity *in vivo* in this vastly interconnected CNS relay.

In line with the associations of higher thalamus ^11^C-ER176 PET uptake with more significant neurodegeneration and disability, the diffuse innate immune activity in the thalamus was higher in progressive pwMS than in relapsing pwMS. While progressive pwMS expectedly were older than relapsing pwMS, we did not observe an age effect as a potential contributor to this difference, further discussed below. Similarly, in a previous study, PET uptake was greater across the brain in secondary progressive pwMS compared with relapsing pwMS.^32^ Moreover, baseline thalamus ^11^C-PK11195 uptake was higher in pwMS who showed disability accumulation within three years.^31^ Because ongoing microglia activity enhances neuronal and axonal loss, the consequent neurodegeneration expectedly associates with progressive symptomatology in pwMS.

In the current study, the patient characteristics of the participant with the highest thalamus ^11^C-ER176 PET uptake in the MS group as well as in the entire cohort further supports the target specificity of ^11^C-ER176 binding in the thalamus reflecting the increased microglia density and disease severity. This pwMS is a 19-year-old male with a disease onset at the age of 17. He has highly active early relapsing MS with an EDSS of 3.5 despite his young age and short disease duration. He was started on natalizumab because he had an active and severe disease with five relapses in 2 years and remarkably high brain and spinal cord lesion burden including thalamus lesions and multiple T1 black holes. However, at the time of PET imaging, he did not have any new or active lesions.

Our study findings, together with previous observations, clearly indicate thalamic microglia changes on ^11^C-ER176 PET as a biomarker of progression in MS. However, this association cannot be claimed as being causative of progressive disease in pwMS. Instead, we propose that thalamic changes act as a lamp post for overall pathology in MS reflected in one critical CNS relay location. Thalamus ^11^C-ER176 PET uptake does not only reflect changes in the CNS from distant connections, as lesions within the thalamus add cumulative burden to elevated thalamus ^11^C-ER176 PET uptake. In the current study, the presence of thalamic lesions resulted in more pronounced thalamus ^11^C-ER176 PET uptake both in the relapsing and progressive pwMS. Therefore, we conclude that not only the diffuse neurodegenerative MS disease mechanisms but also the local thalamic lesional inflammatory activity contribute to the increased innate immune activity in the thalamus in MS. This suggests thalamus ^11^C-ER176 PET uptake as a suitable candidate for reflecting the overall disease burden of collective lesional and nonlesional diffuse CNS damage, regardless of originating from systemic immune activation, CNS innate immune activation or neurodegenerative mechanisms^31,33^ in pwMS.

### Thalamus ^11^C-ER176 PET uptake is influenced by sex

In the current study, in controls, females had higher thalamus ^11^C-ER176 PET uptake than males. Similarly, two animal studies showed higher TSPO PET uptake in females than in males.^34,35^ Moreover, in a retrospective multicenter study of healthy volunteers, females had higher TSPO PET uptake, and this uptake was especially elevated in younger women, which is suggested to contribute to the higher prevalence of autoimmune diseases in younger women.^36^

The higher thalamus ^11^C-ER176 PET uptake in female controls versus male controls did not persist in pwMS, as the values were similar between the two sexes. The nullification of this difference in pwMS may indicate a more significant increase in thalamus ^11^C-ER176 PET uptake in male pwMS than in female pwMS when compared with controls. This higher gradient of difference in men may not be surprising based on our results of more severe and progressive disease being associated with increased thalamus ^11^C-ER176 PET uptake in MS and because men are known to have more severe and progressive MS course than women. Similarly, in another MS study, males had higher thalamic TSPO PET uptake than females,^37^ which supported the higher susceptibility of and acceleration in disability worsening and progression in male pwMS. We cannot make any claims to exact genetic or hormonal mechanisms of the observed sex differences in TSPO PET uptake in multiple studies, but we can conclude that it is necessary to account for sex as a biological variable between study groups in future studies or clinical trials using ^11^C-ER176 PET.^38^

Interestingly, there is no consensus on the association between TSPO PET uptake and age. We did not find an association between thalamus ^11^C-ER176 PET uptake and age in MS nor in controls. Similarly, some of the previous studies showed no correlation between TSPO PET and age in the WM, GM and thalamus in normal aging, Alzheimer’s disease or MS.^39-41^ In contrast, other studies showed that TSPO PET uptake increased with age in multiple regions in normal aging,^36,42,43^ and Alzheimer’s disease,^44^ and in the normal-appearing WM (NAWM) in pwMS.^45^ TSPO PET uptake may be expected to increase with age because both age and microglia, particularly aging microglia, associate with biomarkers of neurodegeneration, which tend to overlap between aging and neurodegenerative diseases.^39-41^ However, sex distributions, sample sizes and variations in mean ages and linear analyses of age may contribute to inconclusive results. Particularly in women, there may be an age-associated threshold for microglia activity that may be further impacted by hormonal status during menopausal transition. Our study falls short in answering the specific question on an age association with thalamic innate immune activation.

### Thalamus ^11^C-ER176 PET uptake is associated with MRI biomarkers of neurodegeneration in patients with multiple sclerosis

In pwMS, higher thalamus ^11^C-ER176 PET uptake correlated with multiple MRI biomarkers of neurodegeneration in the GM and WM, which included thalamus (a critical central CNS relay, which is strongly related to disability and progression), cortical GM and corpus callosum (the largest interhemispheric connection WM tract) that are all typically and immediately affected in MS. The association of higher thalamus ^11^C-ER176 PET uptake with smaller thalamic volume on MRI highlights the contribution of high microglia density and innate immune activity on the gradual neuronal damage and loss in the thalamus.

The contribution of microglia to neurodegeneration and demyelination^46^ was further supported by diffusion MRI, which provided a more detailed assessment of neurodegeneration and demyelination at the microstructural level. Higher thalamus ^11^C-ER176 PET uptake correlated with lower WM integrity on diffusion MRI in thalamus and corpus callosum in pwMS. Similarly, in a previous study, in NAWM, higher TSPO PET uptake was associated with reduced WM integrity (higher MD) in pwMS.^45^

In the current study, we also showed that higher ^11^C-ER176 PET uptake correlated with higher thalamus and corpus callosum neurite (axon + dendrite) damage on NODDI. Using NODDI to evaluate neurite integrity was another novelty of the current study, which provided a more comprehensive and specific assessment of multiple tissue microstructures.^47^

It was previously shown that in MS, higher thalamic TSPO PET uptake correlated with cortical thinning^32^ and, in individuals with cortical ischaemic stroke, TSPO PET uptake was elevated in the ipsilateral thalamus.^48^ These data indicate that the increased microglia activity leads to thalamic changes either as adaptation/compensation or as a signal of neurodegeneration in response to corresponding projections distant from the primary damage.^49,50^ We did not find a correlation between thalamus ^11^C-ER176 PET uptake and cortical thickness. A more benign disease course without sufficient time for cortical atrophy or a smaller representation of progressive pwMS and male patients could have contributed to this lack of association. However, if these explanations were confirmed, our study would also suggest that thalamic microglia changes on ^11^C-ER176 PET likely precede cortical atrophy in MS.

### Thalamus ^11^C-ER176 PET uptake is associated with clinical metrics of disability in patients with multiple sclerosis

Thalamus constantly relays motor and sensory signals and is a key station for numerous networks supporting cognitive function.^51,52^ It was previously shown that using first- or second-generation radioligands, higher TSPO PET uptake in deep GM, NAWM and cortex correlated with disability^32,53,54^ and cognitive impairment in MS.^32^ In line with this, in the current study, in the entire MS cohort, the thalamus ^11^C-ER176 PET uptake was closely associated with characteristic MS-specific disability metrics captured by EDSS and MSFC as well as PASAT.

The persistent microglia activity in the thalamus likely associates with motor disability. It also associates with cognitive impairment, more specifically with deterioration in information processing, attention and working memory measured by PASAT. The correlation between higher thalamus ^11^C-ER176 PET uptake and worse arm/hand function showed a similar direction observed in EDSS, MSFC and PASAT, but this correlation was not statistically significant. Fine motor skills of hand function require sensory coordination, but the relevant motor function may more likely to be represented in higher cervical spinal cord changes and not as well represented in thalamic changes. We also did not find a correlation between thalamus ^11^C-ER176 PET uptake and 25FWT, which is often prominently impacted by motor and cerebellar function, whereas lower extremity motor function and associated motor progression may be more critically related to spinal cord changes and again less represented in thalamic changes. In the current study, it is also possible that the insufficiency of 25FWT in distinguishing minor leg function differences in a cohort with mostly milder motor deficit may have played a role.

### Thalamus ^11^C-ER176 PET as a potential biomarker of progression in multiple sclerosis

Microglia are sentinel cells of the CNS, which continuously monitor their microenvironment and quickly respond to threats.^55^ However, interestingly, microglia become a threat to the CNS when they are overactivated. Inflammatory microglia promote neurodegeneration by inducing neurotoxicity through inflammatory cytokines, iron deposition and oxidative stress,^56^ and they are mediated by multiple proteins including BTK.^6^

The increase in innate immune activity plays a key role in progression in MS^1^ and the microglia PET can detect the persistently active microglia density.^57^ The widespread pathology of MS is reflected in the thalamus, a key CNS relay that is affected early in the disease course. Our study supports that microglia PET can evaluate the widespread brain pathology associated with progressive MS, and thalamus is an optimal location to quantify increased innate immune activity in MS.

Our study findings show how continuous and increased innate immune system activity contributes to other disease mechanisms leading to tissue loss, disability and, ultimately, progression in MS while accounting for the moderating effect of sex. They also suggest ^11^C-ER176 PET as a candidate biomarker in quantifying treatment outcomes in clinical trials targeting inflammatory microglia, such as BTK inhibitors, an emerging group of therapeutics for relapsing and progressive MS^58^ as well as in future novel therapy trials to delay or stop progression in MS.^59^

Regarding current DMTs, in pwMS, we did not observe any differences in thalamus ^11^C-ER176 PET uptake among high-efficacy DMT, moderate-efficacy DMT and no-DMT groups. These findings may suggest that there is no significant overall DMT effect on thalamus ^11^C-ER176 PET uptake. Sample size, age and sex differences might have contributed to this lack of difference in thalamus ^11^C-ER176 PET uptake between the DMT groups. Furthermore, expectedly, no individual DMT effect was detected on thalamus ^11^C-ER176 PET uptake, possibly due to not having enough power. Similarly, in a previous study, longitudinally, no change was observed in the thalamus TSPO PET uptake after 6 months of natalizumab treatment in pwMS.^60^

This study had some limitations. A group of participants did not have TSPO single nucleotide polymorphism analysis. However, the low-affinity binding is rare and only 2 of the 27 participants with DNA available in our study had low-affinity binding. However, the thalamus ^11^C-ER176 PET uptake of these participants (1.294 SUVR and 1.261 SUVR) were higher than the mean thalamus ^11^C-ER176 PET uptake of the whole cohort (1.194 ± 0.078 SUVR). This further confirms that the third-generation radioligand ^11^C-ER176 is significantly less affected by affinity than second-generation radioligands. Binding measurements must still be corrected post-hoc for genotype, particularly for following individuals in a longitudinal analysis in a clinical trial setting. However, this should not impact our results as discussed above. Furthermore, DMT use in the MS group varied because pwMS were prospectively enrolled from the MS clinic and were not randomized in a clinical trial setting.

Based on our data, we can conclude that thalamus ^11^C-ER176 PET is a valid molecular imaging biomarker of progression in MS, reflecting the present global disease severity stemming from distant and diffuse neurodegenerative changes as well as local lesional damage. It remains to be seen if glial changes precede or parallel neurodegenerative changes, which would require serial TSPO PET studies. Such a longitudinal approach would be similar to amyloid-β PET studies, which demonstrated that amyloid-β pathology starts early in Alzheimer's disease, before the emergence of cognitive symptoms.^61^

## Data Availability

The data that support the findings of this study are available from the corresponding author upon reasonable request.

## References

[1] Airas L, Yong VW. Microglia in multiple sclerosis—Pathogenesis and imaging. Curr Opin Neurol. 2022;35(3):299–306. 35674072 10.1097/WCO.0000000000001045

[2] Ikawa M, Lohith TG, Shrestha S, et al 11C-ER176, a radioligand for 18-kDa translocator protein, has adequate sensitivity to robustly image all three affinity genotypes in human brain. J Nucl Med. 2017;58(2):320–325. 27856631 10.2967/jnumed.116.178996 PMC5288742

[3] Azevedo CJ, Overton E, Khadka S, et al Early CNS neurodegeneration in radiologically isolated syndrome. Neurol Neuroimmunol Neuroinflamm. 2015;2(3):e102. 25884012 10.1212/NXI.0000000000000102 PMC4396526

[4] Bauckneht M, Capitanio S, Raffa S, et al Molecular imaging of multiple sclerosis: From the clinical demand to novel radiotracers. EJNMMI Radiopharm Chem. 2019;4(1):6. 31659498 10.1186/s41181-019-0058-3 PMC6453990

[5] Kramer J, Bar-Or A, Turner TJ, Wiendl H. Bruton tyrosine kinase inhibitors for multiple sclerosis. Nat Rev Neurol. 2023;19(5):289–304. 37055617 10.1038/s41582-023-00800-7 PMC10100639

[6] Gruber RC, Wirak GS, Blazier AS, et al BTK regulates microglial function and neuroinflammation in human stem cell models and mouse models of multiple sclerosis. Nat Commun. 2024;15(1):10116. 39578444 10.1038/s41467-024-54430-8 PMC11584639

[7] Roberts RO, Geda YE, Knopman DS, et al The Mayo Clinic study of aging: Design and sampling, participation, baseline measures and sample characteristics. Research support, N.I.H., ExtramuralResearch support, non-U.S. Gov't. Neuroepidemiology. 2008;30(1):58–69. 18259084 10.1159/000115751 PMC2821441

[8] Thompson AJ, Banwell BL, Barkhof F, et al Diagnosis of multiple sclerosis: 2017 revisions of the McDonald criteria. Lancet Neurol. 2018;17(2):162–173. 29275977 10.1016/S1474-4422(17)30470-2

[9] Lebrun-Frenay C, Okuda DT, Siva A, et al The radiologically isolated syndrome: Revised diagnostic criteria. Brain. 2023;146(8):3431–3443. 36864688 10.1093/brain/awad073 PMC11004931

[10] Brownlee WJ, Vidal-Jordana A, Shatila M, et al Towards a unified set of diagnostic criteria for multiple sclerosis. Ann Neurol. 2024;97(3): 571–582. 39605172 10.1002/ana.27145 PMC11831880

[11] Kurtzke JF . Rating neurologic impairment in multiple sclerosis: An expanded disability status scale (EDSS). Neurology. 1983;33(11):1444–1452. 6685237 10.1212/wnl.33.11.1444

[12] Fischer JS, Rudick RA, Cutter GR, Reingold SC. The multiple sclerosis functional composite measure (MSFC): An integrated approach to MS clinical outcome assessment. National MS society clinical outcomes assessment task force. Mult Scler. 1999;5(4):244–250. 10467383 10.1177/135245859900500409

[13] Schwarz CG, Gunter JL, Ward CP, et al The mayo clinic adult life span template: Better quantification across the life span. Alzheimer's & Dementia. 2017;13(7):P93–P94.

[14] Schwarz CG, Gunter JL, Wiste HJ, et al A large-scale comparison of cortical thickness and volume methods for measuring Alzheimer's disease severity. Neuroimage Clin. 2016;11:802–812. 28050342 10.1016/j.nicl.2016.05.017 PMC5187496

[15] Caruyer E, Lenglet C, Sapiro G, Deriche R. Design of multishell sampling schemes with uniform coverage in diffusion MRI. Magn Reson Med. 2013;69(6):1534–1540. 23625329 10.1002/mrm.24736 PMC5381389

[16] Veraart J, Novikov DS, Christiaens D, Ades-Aron B, Sijbers J, Fieremans E. Denoising of diffusion MRI using random matrix theory. Neuroimage. 2016;142:394–406. 27523449 10.1016/j.neuroimage.2016.08.016 PMC5159209

[17] Andersson JLR, Sotiropoulos SN. An integrated approach to correction for off-resonance effects and subject movement in diffusion MR imaging. Neuroimage. 2016;125:1063–1078. 26481672 10.1016/j.neuroimage.2015.10.019 PMC4692656

[18] Garyfallidis E, Brett M, Amirbekian B, et al Dipy, a library for the analysis of diffusion MRI data. Front Neuroinform. 2014;8:8. 24600385 10.3389/fninf.2014.00008 PMC3931231

[19] Zhang H, Schneider T, Wheeler-Kingshott CA, Alexander DC. NODDI: Practical in vivo neurite orientation dispersion and density imaging of the human brain. Neuroimage. 2012;61(4):1000–1016. 22484410 10.1016/j.neuroimage.2012.03.072

[20] Daducci A, Canales-Rodriguez EJ, Zhang H, Dyrby TB, Alexander DC, Thiran JP. Accelerated microstructure imaging via convex optimization (AMICO) from diffusion MRI data. Neuroimage. 2015;105:32–44. 25462697 10.1016/j.neuroimage.2014.10.026

[21] Schwarz CG, Senjem ML, Gunter JL, et al Optimizing PiB-PET SUVR change-over-time measurement by a large-scale analysis of longitudinal reliability, plausibility, separability, and correlation with MMSE. Neuroimage. 2017;144(Pt A):113–127. 27577718 10.1016/j.neuroimage.2016.08.056 PMC5183471

[22] Kreisl WC, Fujita M, Fujimura Y, et al Comparison of [(11)C]-(R)-PK 11195 and [(11)C]PBR28, two radioligands for translocator protein (18 kDa) in human and monkey: Implications for positron emission tomographic imaging of this inflammation biomarker. Neuroimage. 2010;49(4):2924–2932. 19948230 10.1016/j.neuroimage.2009.11.056 PMC2832854

[23] Kreisl WC, Kim MJ, Coughlin JM, Henter ID, Owen DR, Innis RB. PET imaging of neuroinflammation in neurological disorders. Lancet Neurol. 2020;19(11):940–950. 33098803 10.1016/S1474-4422(20)30346-X PMC7912433

[24] Viviano M, Barresi E, Simeon FG, et al Essential principles and recent progress in the development of TSPO PET ligands for neuroinflammation imaging. Curr Med Chem. 2022;29(28):4862–4890. 35352645 10.2174/0929867329666220329204054 PMC10080361

[25] Cumbers GA, Harvey-Latham ED, Kassiou M, Werry EL, Danon JJ. Emerging TSPO-PET radiotracers for imaging neuroinflammation: A critical analysis. Semin Nucl Med. 2024;54(6):856–874. 39477764 10.1053/j.semnuclmed.2024.09.007

[26] Vowinckel E, Reutens D, Becher B, et al PK11195 binding to the peripheral benzodiazepine receptor as a marker of microglia activation in multiple sclerosis and experimental autoimmune encephalomyelitis. J Neurosci Res. 1997;50(2):345–353. 9373043 10.1002/(SICI)1097-4547(19971015)50:2<345::AID-JNR22>3.0.CO;2-5

[27] Banati RB, Myers R, Kreutzberg GW. PK (‘peripheral benzodiazepine’)–binding sites in the CNS indicate early and discrete brain lesions: Microautoradiographic detection of [3H]PK11195 binding to activated microglia. J Neurocytol. 1997;26(2):77–82. 9181482 10.1023/a:1018567510105

[28] Banati RB, Newcombe J, Gunn RN, et al The peripheral benzodiazepine binding site in the brain in multiple sclerosis: Quantitative in vivo imaging of microglia as a measure of disease activity. Brain. 2000;123(Pt 11):2321–2337. 11050032 10.1093/brain/123.11.2321

[29] Fujita M, Kobayashi M, Ikawa M, et al Comparison of four (11)C-labeled PET ligands to quantify translocator protein 18 kDa (TSPO) in human brain: (R)-PK11195, PBR28, DPA-713, and ER176-based on recent publications that measured specific-to-non-displaceable ratios. EJNMMI Res. 2017;7(1):84. 29038960 10.1186/s13550-017-0334-8 PMC5643834

[30] Zanotti-Fregonara P, Pascual B, Veronese M, et al Head-to-head comparison of (11)C-PBR28 and (11)C-ER176 for quantification of the translocator protein in the human brain. Eur J Nucl Med Mol Imaging. 2019;46(9):1822–1829. 31152207 10.1007/s00259-019-04349-w

[31] Misin O, Matilainen M, Nylund M, et al Innate immune cell-related pathology in the thalamus signals a risk for disability progression in multiple sclerosis. Neurol Neuroimmunol Neuroinflamm. 2022;9(4):e1182. 35581004 10.1212/NXI.0000000000001182 PMC9128041

[32] Herranz E, Gianni C, Louapre C, et al Neuroinflammatory component of gray matter pathology in multiple sclerosis. Ann Neurol. 2016;80(5):776–790. 27686563 10.1002/ana.24791 PMC5115951

[33] Kipp M, Wagenknecht N, Beyer C, Samer S, Wuerfel J, Nikoubashman O. Thalamus pathology in multiple sclerosis: From biology to clinical application. Cell Mol Life Sci. 2015;72(6):1127–1147. 25417212 10.1007/s00018-014-1787-9 PMC11113280

[34] Schwarz JM, Sholar PW, Bilbo SD. Sex differences in microglial colonization of the developing rat brain. J Neurochem. 2012;120(6):948–963. 22182318 10.1111/j.1471-4159.2011.07630.x PMC3296888

[35] Flurkey K, Currer JM, Harrison DM. Mouse models in aging research, chap 20. In: Fox JG, Davisson MT, Quimby FW, Barthold SW, Newcomer CE, Smith AL, eds. The mouse in biomedical research. Second ed. Elsevier; 2007:637–672.

[36] Tuisku J, Plaven-Sigray P, Gaiser EC, et al Effects of age, BMI and sex on the glial cell marker TSPO—A multicentre [(11)C]PBR28 HRRT PET study. Eur J Nucl Med Mol Imaging. 2019;46(11):2329–2338. 31363804 10.1007/s00259-019-04403-7 PMC6717599

[37] Laaksonen S, Saraste M, Nylund M, et al Sex-driven variability in TSPO-expressing microglia in MS patients and healthy individuals. Front Neurol. 2024;15:1352116. 38445263 10.3389/fneur.2024.1352116 PMC10913932

[38] Nathoo N, Neyal N, Kantarci OH, Zeydan B. Imaging phenotypic differences in multiple sclerosis: At the crossroads of aging, sex, and race. Front Glob Womens Health. 2024;5:1412482. 39006184 10.3389/fgwh.2024.1412482 PMC11245741

[39] Debruyne JC, Versijpt J, Van Laere KJ, et al PET visualization of microglia in multiple sclerosis patients using [11C]PK11195. Eur J Neurol. 2003;10(3):257–264. 12752399 10.1046/j.1468-1331.2003.00571.x

[40] Yasuno F, Ota M, Kosaka J, et al Increased binding of peripheral benzodiazepine receptor in Alzheimer's disease measured by positron emission tomography with [11C]DAA1106. Biol Psychiatry. 2008;64(10):835–841. 18514164 10.1016/j.biopsych.2008.04.021

[41] Suridjan I, Rusjan PM, Voineskos AN, et al Neuroinflammation in healthy aging: A PET study using a novel translocator protein 18 kDa (TSPO) radioligand, [(18)F]-FEPPA. Neuroimage. 2014;84:868–875. 24064066 10.1016/j.neuroimage.2013.09.021 PMC6283059

[42] Paul S, Gallagher E, Liow JS, et al Building a database for brain 18 kDa translocator protein imaged using [(11)C]PBR28 in healthy subjects. J Cereb Blood Flow Metab. 2019;39(6):1138–1147. 29749279 10.1177/0271678X18771250 PMC6547185

[43] Schuitemaker A, van der Doef TF, Boellaard R, et al Microglial activation in healthy aging. Neurobiol Aging. 2012;33(6):1067–1072. 21051106 10.1016/j.neurobiolaging.2010.09.016

[44] Cagnin A, Brooks DJ, Kennedy AM, et al In-vivo measurement of activated microglia in dementia. Lancet. 2001;358(9280):461–467. 11513911 10.1016/S0140-6736(01)05625-2

[45] Rissanen E, Tuisku J, Vahlberg T, et al Microglial activation, white matter tract damage, and disability in MS. Neurol Neuroimmunol Neuroinflamm. 2018;5(3):e443. 29520366 10.1212/NXI.0000000000000443 PMC5840890

[46] Kooistra SM, Schirmer L. Multiple sclerosis: Glial cell diversity in time and space. Glia. 2025;73(3):574–590. 39719685 10.1002/glia.24655 PMC11784844

[47] Caranova M, Soares JF, Batista S, Castelo-Branco M, Duarte JV. A systematic review of microstructural abnormalities in multiple sclerosis detected with NODDI and DTI models of diffusion-weighted magnetic resonance imaging. Magn Reson Imaging. 2023;104:61–71. 37775062 10.1016/j.mri.2023.09.010

[48] Pappata S, Levasseur M, Gunn RN, et al Thalamic microglial activation in ischemic stroke detected in vivo by PET and [11C]PK1195. Neurology. 2000;55(7):1052–1054. 11061271 10.1212/wnl.55.7.1052

[49] Kolasinski J, Stagg CJ, Chance SA, et al A combined post-mortem magnetic resonance imaging and quantitative histological study of multiple sclerosis pathology. Brain. 2012;135(Pt 10):2938–2951. 23065787 10.1093/brain/aws242 PMC3470716

[50] Mahajan KR, Nakamura K, Cohen JA, Trapp BD, Ontaneda D. Intrinsic and extrinsic mechanisms of thalamic pathology in multiple sclerosis. Ann Neurol. 2020;88(1):81–92. 32286701 10.1002/ana.25743 PMC8291218

[51] Torrico TJ, Munakomi S. Neuroanatomy, thalamus. StatPearls; 2024. 31194341

[52] Herrero MT, Barcia C, Navarro JM. Functional anatomy of thalamus and basal ganglia. Childs Nerv Syst. 2002;18(8):386–404. 12192499 10.1007/s00381-002-0604-1

[53] Politis M, Giannetti P, Su P, et al Increased PK11195 PET binding in the cortex of patients with MS correlates with disability. Neurology. 2012;79(6):523–530. 22764258 10.1212/WNL.0b013e3182635645 PMC3413767

[54] Sucksdorff M, Matilainen M, Tuisku J, et al Brain TSPO-PET predicts later disease progression independent of relapses in multiple sclerosis. Brain. 2020;143(11):3318–3330. 33006604 10.1093/brain/awaa275 PMC7719021

[55] Nimmerjahn A, Kirchhoff F, Helmchen F. Resting microglial cells are highly dynamic surveillants of brain parenchyma in vivo. Science. 2005;308(5726):1314–1318. 15831717 10.1126/science.1110647

[56] Faissner S, Plemel JR, Gold R, Yong VW. Progressive multiple sclerosis: From pathophysiology to therapeutic strategies. Nat Rev Drug Discov. 2019;18(12):905–922. 31399729 10.1038/s41573-019-0035-2

[57] Airas L, Nylund M, Rissanen E. Evaluation of microglial activation in multiple sclerosis patients using positron emission tomography. Front Neurol. 2018;9:181. 29632509 10.3389/fneur.2018.00181 PMC5879102

[58] Airas L, Bermel RA, Chitnis T, et al A review of bruton's tyrosine kinase inhibitors in multiple sclerosis. Ther Adv Neurol Disord. 2024;17:17562864241233041. 38638671 10.1177/17562864241233041 PMC11025433

[59] Bodini B, Tonietto M, Airas L, Stankoff B. Positron emission tomography in multiple sclerosis—Straight to the target. Nat Rev Neurol. 2021;17(11):663–675. 34545219 10.1038/s41582-021-00537-1

[60] Kaunzner UW, Kang Y, Monohan E, et al Reduction of PK11195 uptake observed in multiple sclerosis lesions after natalizumab initiation. Mult Scler Relat Disord. 2017;15:27–33. 28641769 10.1016/j.msard.2017.04.008

[61] Jack CR Jr, Andrews SJ, Beach TG, et al Revised criteria for the diagnosis and staging of Alzheimer's disease. Nat Med. 2024;30(8):2121–2124. 38942991 10.1038/s41591-024-02988-7 PMC11630478

